# Relationship of Glucocorticoid Receptor Expression in Peripheral Blood Mononuclear Cells and the Cochlea of Guinea Pigs and Effects of Dexamethasone Administration

**DOI:** 10.1371/journal.pone.0056323

**Published:** 2013-02-07

**Authors:** Ling Lu, Yanhong Dai, Xiaoping Du, Wandong She, Xiuling Zhang, Qin Wu, Wenjie Yuan, Feng Chen

**Affiliations:** 1 Department of Otolaryngology-Head and Neck Surgery, Nanjing Drum Tower Hospital, Nanjing University Medical School, Nanjing, China; 2 Hough Ear Institute, Oklahoma City, Oklahoma, United States of America; 3 Jiangyin People's Hospital, Southeast University Medical College, Jiangyin, Jiangsu Province, China; 4 Microbiology Laboratory, Nanjing University Medical School, Nanjing, China; Universidade Federal do Rio de Janeiro, Brazil

## Abstract

**Background:**

Glucocorticoids (GCs) are widely used to treat sudden sensorineural hearing loss (SSNHL) and significantly improve hearing. However, GC insensitivity has been observed in some patients of SSNHL.

**Objective:**

To study the correlation between GR expression in peripheral blood mononuclear cells (PBMCs) and in the cochlea of guinea pigs at mRNA and protein levels.

**Methods:**

One group of guinea pigs received dexamethasone (10 mg/kg/day) intraperitoneally for 7 consecutive days (dexamethasone group), and another group of guinea pigs received normal saline (control group). Real time PCR and Western blotting were used to detect the expression of GR mRNA and GR protein in PBMCs and the cochleae.

**Results:**

The GR mRNA and GR protein were detected in both PBMCs and the cochlear tissue of guinea pigs. GR mRNA and GR protein levels in PBMCs were positively correlated with those in the cochlea. The expression of GR mRNA and GR protein was significantly increased in the dexamethasone group compared to the control group.

**Conclusions:**

Levels of GR mRNA and GR protein in the PBMCs were positively correlated with those in the cochlea of guinea pigs. Systemic dexamethasone treatment can significantly up-regulate GR expression in PBMCs and in the cochlea. Measurement of the GR level in PBMCs could be used as an indicator of GR level in the cochlea.

## Introduction

The cause of sudden sensorineural hearing loss (SSNHL) is still unclear, although various infective, vascular, and immune causes have been proposed [1]. Glucocorticoids (GCs) are widely used to treat SSNHL and significantly improve hearing [1], [2]. GCs could inhibit several inflammatory mediators and increase cochlear blood flow to prevent hair cell damage caused by inflammation and ischemia in the inner ear [3]–[5]. However, GC insensitivity has been observed in some patients of SSNHL, as well as in patients with severe asthma, rheumatoid arthritis (RA), ulcerative colitis and systemic lupus erythematosus (SLE) [6]–[11]. The mechanisms of GC insensitivity are still unclear.

The actions of GCs are predominantly mediated through their common receptor, the glucocorticoid receptor (GR). As a DNA-binding transcription factor of the nuclear receptor superfamily, GR is ubiquitously expressed throughout the body, including the cochlea [12]. Upon binding to GCs, the cytoplasmic GR undergoes a conformational change, and the resulting activated GR translocates from the cell cytoplasm into the nucleus. Once in the nucleus, ligand-bound GR interacts with regulatory elements in the genome to induce or repress transcription of hundreds of target genes to control regulatory networks in fetal development, metabolism, cognition and inflammation [13]–[15].

Rarey and Curtis first detected GR in the human inner ear using enzyme-linked immunosorbent assay (ELISA) [16]. They found that the highest concentration of GR protein was in the spiral ligament tissues, and the lowest in the saccule [16]. A positive correlation has been reported between GR expression in peripheral blood mononuclear cells (PBMCs) and renal intrinsic cells, and the expression level of GR β in PBMCs and the renal cells may be related to the GC-sensitivity in patients with primary nephrotic syndrome [17]. Barnes and Adack have also pointed out that GRβ plays an important role in some GR-resistance inflammatory diseases [18]. Therefore, we hypothesized that GR level in the cochlea of SSNHL patients may be related to GR sensitivity. However, the human cochlea is located deep within the temporal bone. As a result, it is impractical to directly screen for cochlear GR content in patients diagnosed with SSNHL, and thus, an indirect method for detecting the GR level in the cochlea is needed. In the present study, we analyzed the expression levels of GR mRNA and GR protein in PBMCs and cochleae from guinea pigs to ascertain whether PBMCs could serve as a peripheral surrogate for ascertaining GR expression levels in the cochleae from the same animal and investigated whether the systemic administration of dexamethasone impacted GR expression in cochleae and PBMCS synchronistically.

## Materials and Methods

### Animals

Healthy albino guinea pigs (250 to 300 g body weight, male and female) with normal auricle reflex were purchased from Qing Longshan Animal Breeding Laboratory (Nanjing, China) and housed in the Animal Center of Nanjing Drum Tower Hospital, Nanjing University Medical School. All experimental procedures and animal handling were approved by and complied with the requirements of the Animal Care and Use Committee of Nanjing University under national ethics guidelines. The middle ears were examined with electric auriscopy. The animals were randomly divided into two groups: dexamethasone group (DG) and control group (CG). Dexamethasone (10 mg/kg/day, 5 mg/ml, Wuhu Kangqi Pharmaceutical Co., LTD, Wuhu, China) was intraperitoneally injected for 7 consecutive days in 15 guinea pigs in the DG. The same volume of normal physiological saline was intraperitoneally injected in 17 guinea pigs in the CG.

### PBMCs and cochlear tissue isolation

Guinea pigs were anesthetized with ketamine (75 mg/kg, IM) one day after dexamethasone administration. Blood was collected from the venae angularis. The animals were then euthanized by decapitation, and the cochleae were immediately dissected out and placed in ice-cold saline. The soft tissue (including the basilar membrane and the lateral wall) of each cochlear duct was harvested under a dissection microscope and then stored at −80 °C prior to RNA and protein isolation. PBMCs were separated using Ficoll-Paque Plus gradient centrifugation according to the manufacturer's protocol (Amersham Biosciences, Uppsala, Sweden) and then stored at −80 °C prior to RNA and protein isolation.

### Quantitative real-time PCR

Total RNA was extracted from PBMCs and the soft tissue from the left cochlea of each guinea pig using Trizol reagent (Invitrogen, CO., Carlsbad, CA, USA) according to the manufacturer's protocol. The RNA quality was assessed by an Agilent Bioanalyzer 2100 (Santa Clara, CA, USA). Reverse transcription of RNA was carried out with the PrimeScript RT-PCR Kit (TaKaRa, Otsu, Shiga, Japan) according to the manufacturer's protocol using oligo dT Primers to generate a high fidelity cDNA pool, bearing initiation sites for T7 RNA polymerase at their 3′ ends. The Applied Biosystems 7500 real-time PCR system (Applied Biosystems, Carlsbad, CA, USA) was used to perform quantitative real-time PCR (QRT-PCR). A parallel QRT-PCR reaction was performed with β-actin as an internal control. QRT-PCR of each sample was run in triplicate for technical control and statistical analysis. The PCR primers were designed by Primer 5.0 software and synthesized by Invitrogen CO. (Carlsbad, CA, USA): GR forward—5′-CTGCATCTTCACCCTCAC-3′ and reverse—5′-CTTTACATTGCCACCATT-3′; β-actin forward—5′-CTTTGCTGCGTTACACCC-3′ and reverse—5′-GTCACCTTCACCGTTCCA-3′. The thermal cycle conditions included a 30 sec initial set-up at 95 °C followed by 40 cycles of 5 sec denaturing at 95 °C and 20 sec annealing/extension at 60 °C. The results of each plate were analyzed using PRISM software (Applied Biosystems, Carlsbad, CA, USA) to calculate the C_T_ value of each well and to compare the C_T_ values measured for each target transcript with those measured for the internal controls.

### Western blot analysis

Protein in each right cochlear soft tissue was extracted with lysis buffer (20 mM Tris HCl (pH 7.5–8.0), 150 mM NaCl, 0.5% sodium deoxycolate, 1% Triton ×100, 0.1 sodium dodecyl sulfate (SDS), 1 mM EDTA, 1 mM phenylmethylsulphonyl fluoride (PMSF), and protease inhibitor cocktail) at 4 °C for 10 min. PBMCs were lysed with CellLytic M cell lysis buffer (Sigma, St Louis, MO, USA) at 4 °C for 10 min. The cochlear soft tissue and PBMC homogenates were centrifuged at 14,000 RPM for 10 min at 4 °C. Protein concentration in the supernatant was determined by the bicinchoninic acid (BCA) protein assay (Rockford, IL, USA). Approximately 50 µg of the supernatant was separated by SDS-polyacrylamide gel electrophoresis (PAGE) and electrophoretically transferred onto a polyvinylidene fluoride (PVDF) membrane. The membrane was incubated in a blocking buffer containing 5% nonfat dry milk in TBS for 1 hour and then incubated with rabbit anti-GR IgG (1∶200, Beijing Boisynthesis Biotechnology CO. Beijing, China) or with anti-GAPDH IgG (1∶1000, Bioworld Technology, Minneapolis, MN, USA) diluted in TBS-T buffer containing 2% milk at 4 °C overnight. The membranes were washed three times with TBS-T and then incubated with goat anti-rabbit IgG (peroxidase conjugated, 1∶5000, Santa Cruz, CA, USA) in TBS-T buffer containing 3% milk for 2 hours at room temperature. The membranes were washed again with TBS-T and visualized with the ECL western blotting detection reagent (GE Healthcare, Rockford, IL, USA).

### Data Analysis

All statistical analyses were conducted using SPSS16.0 software (IBM, Armonk, NY, USA). The measurement data were expressed as the mean±SEM and were analyzed by unpaired Student *t*-test. The Mann-Whitney rank-sum test was used when the data were unevenly distributed. The correlation of GR levels between PBMCs and cochlea were analyzed by Pearson correlation analysis. Statistical significance was set at *p*<0.05.

## Results

To test the hypothesis that GR levels in the cochlea may be related to that in PBMCs, quantitative real-time PCR and western blots were used as complementary approaches to detect the expression levels of GR mRNA and GR protein in both PBMCs and cochleae from 32 cases of healthy guinea pigs. Comparative analyses of GR expression levels in DG and CG were then conducted in order to investigate how the systemic administration of the glucocorticoid, dexamethasone, impacted GR expression in PBMCs and cochleae of guinea pigs.

### GR mRNA expression in PBMCs and the cochlea

GR mRNA was expressed in both PBMCs and the cochlear soft tissue of guinea pigs. The expression levels of GR mRNA in PBMCs and cochlea were significantly increased in the presence of dexamethasone by 2.389±0.108 and 3.052±0.194 fold, respectively, (*P*_PBMCs_ = 0.0027 and *P*_cochlea_ = 0.0085, respectively). This result indicates that GR expression levels in PBMCs and cochlear soft tissue exhibit a synchronized increase after short-term systemic administration of dexamethasone.

**Figure 1:**
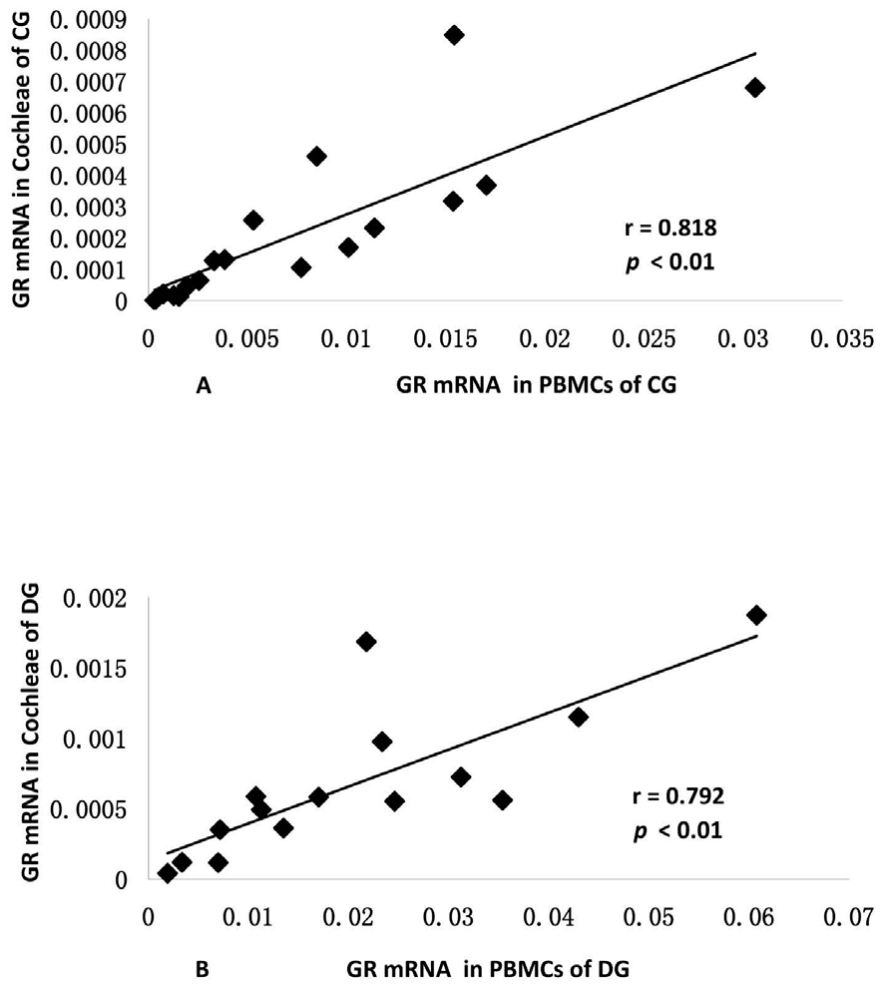
The expression level of GR mRNA in PBMCs was significantly correlated with that in cochlea from both the control group (Fig. 1A, r = 0.818, *p*<0.01) and the dexamethasone group (Fig. 1B, r = 0.792, *p*<0.01).

The correlation between GR levels in PBMCs and in cochleae was then analyzed in both the control and dexamethasone groups. The expression level of GR mRNA in PBMCs was significantly correlated with that measured in the cochlea in the control group (Fig. 1A, r = 0.818, *p*<0.01). Moreover, the induction pattern of GR mRNA expression in cochleae was positively correlated with that observed PMBCs in the dexamethasone group (Fig. 1B, r = 0.792, *p*<0.01).

### GR protein expression in PBMCs and the cochlea

**Figure 2:**
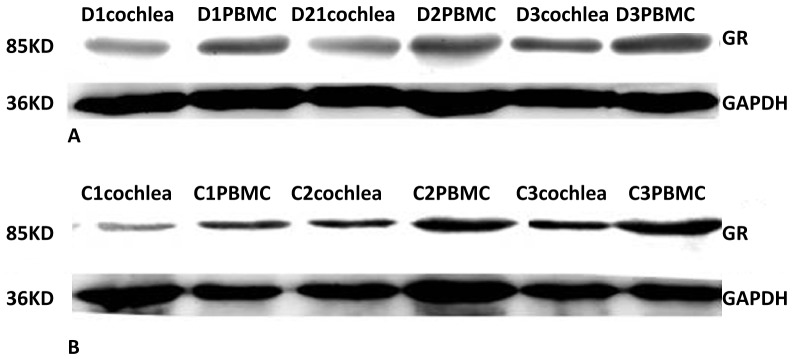
Examples of the GR protein band in PBMCs and cochleae of representative guinea pigs from both the control group (Fig. 2A, C1, C2 and C3) and the dexamethasone group (Fig. 2B, D1, D2 and D3). GAPDH was used as an endogenous control.

**Figure 3:**
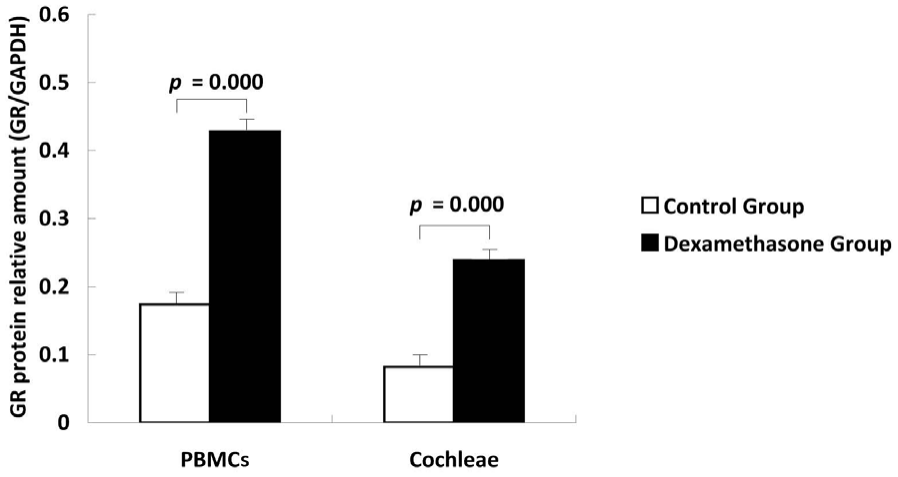
Relative GR protein levels (GR/GAPDH) in PBMCs and cochleae from both the control group and the dexamethasone group. Dexamethasone administration synchronously up-regulated the GR protein levels in PBMCs and Cochleae in guinea pigs.

Western blot analysis confirmed that GR protein was expressed in both PBMCs and cochleae from untreated or dexamethasone-treated guinea pigs (Fig. 2). The relative GR protein levels (GR/GAPDH ratio) in matched PBMCs and cochleae from animals in both treatment groups were then compared for statistical analyses. In PBMCs, the expression level of GR protein was significantly increased in the dexamethasone group compared to the control group (Fig. 3, t = 9.602, *p*<0.01). This same relationship was observed in the cochleae (Fig. 3, t = 9.759, *p*<0.01). These results suggest that GR protein levels were increased simultaneously in PBMCs and cochlear tissue after short-term systemic use of dexamethasone compared to the control group.

**Figure 4:**
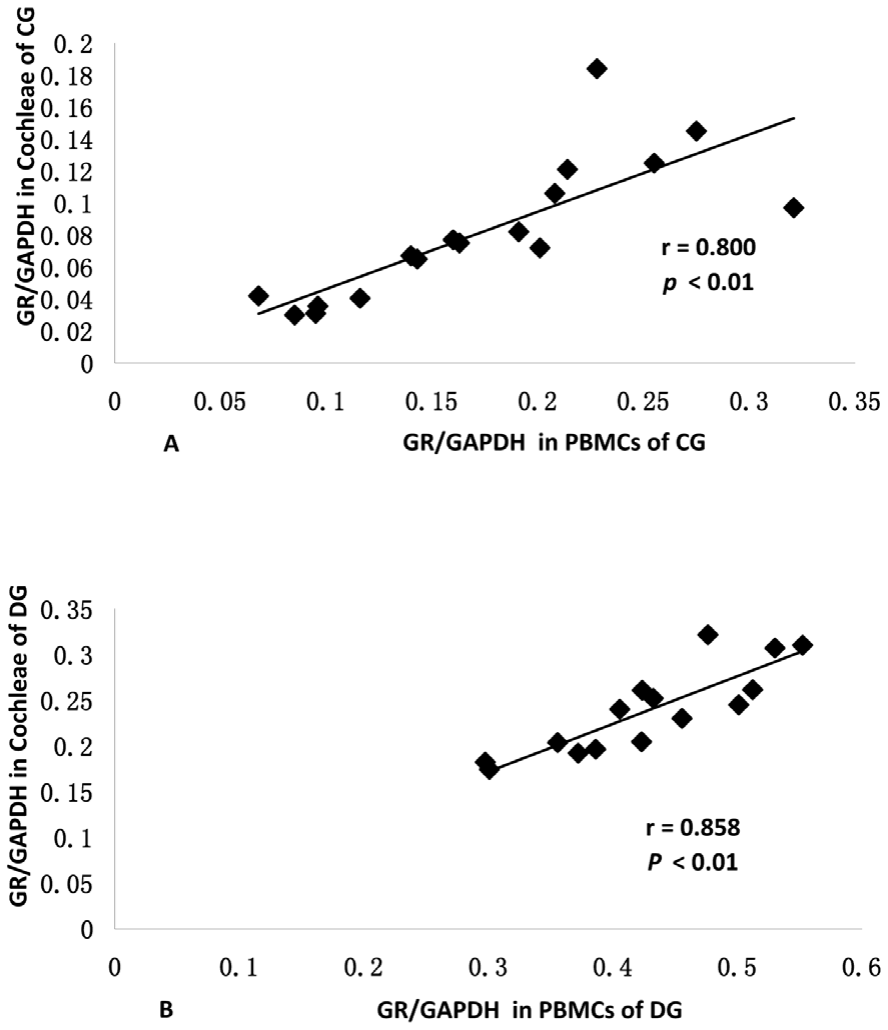
The GR protein level in PBMCs was significantly correlated with that observed in the cochleae from both the control group (**Fig? 4A**, r = 0.800, *p*<0.01) and the dexamethasone group (**Fig? 4B**, r = 0.858, *p*<0.01).

The correlation between GR protein levels in PBMCs and in cochleae was then analyzed in the control group and dexamethasone group to determine whether PBMCs could serve as a peripheral reporter for changes in the expression level of GR manifested in the cochleae upon dexamethasone treatment. As shown in Fig. 4, the GR protein level in PBMCs was significantly correlated with that measured in cochlea in both the control group (Fig. 4A, r = 0.800) and in the dexamethasone group (Fig. 4B, r = 0.858), demonstrating that GR expression is under similar regulatory control in both PBMCs and cochleae.

## Discussion

The interaction between GCs and their receptors is characterized by high selectivity and saturation. The efficacy of GCs may be closely associated with the expression level of GRs, which is confirmed in some diseases [19]. We hypothesized that the level of GRs in the cochlea might be an important factor affecting the efficacy of GCs in treating SSNHL. In the present study, we found GR was expressed in both PBMCs and cochleae in guinea pigs, although its expression levels varied between test animals. Our results demonstrated that the expression level of GR mRNA and GR protein in PBMCs was positively correlated with that in the cochlea of normal healthy guinea pigs, and the GR level in PBMCs and the cochlea of healthy guinea pigs increased simultaneously after a short-term dexamethasone administration. This relationship has been also observed between PBMCs and the kidney [17]. Therefore, the expression level of GR in PBMCs may be a predictive tool for distally assessing GR level in the cochlea. This finding establishes a proof of principle that the expression level of GR in PBMCs may be used to gauge predicted GR sensitivity in SSNHL, prognostically classifying patients who may be resistant to GCs, thus proactively implementing alternative therapies to avoid unnecessary side effects of GCs. However, GC insensitivity can also be attributable to loss of function mutations in the sequence of GR [20]–[22]. Therefore, sequencing analyses to probe for mutations in GR may be a beneficial complementary diagnostic approach in patients with SSNHL.

The GR level in PBMCs and the cochlea of healthy guinea pigs increased simultaneously after a short-term dexamethasone administration, indicating that short-term systemic steroid treatment could positively regulate the expression of GR. Normally, GR expression is down-regulated by glucocorticoids as the result of a feedback protective mechanism. However, up-regulation of GR has been observed in nasal polyps after short term (2 weeks) oral and intranasal glucocorticoid treatment[23]. Under these conditions, up-regulation of GR may enhance the physiological effects of GCs and improve the individual stress responsiveness. Up-regulation of GR mRNA in the inferior colliculus after noise exposure may be associated with stress-induced transient hypersensitivity of the auditory system [24]. However, the endogenous GCs in guinea pigs produced by stress under pathological states, as well as long-term GC application, could down-regulate the expression of GR [25]. The results of the present study were obtained following the administration of a high dose of dexamethasone in normal, healthy guinea pigs. It should also be noted that dexamethasone has been shown to be a potent inhibitor of hypothalamic-pituitary-adrenal (HPA) axis [26]. Therefore, additional studies that address the changes of GR expression levels after short-term systemic use of low doses of GCs under normal and pathological conditions and the effect of dexamethasone on normal plasmatic GC levels are still needed.

In Humans, the GR has two major isoforms, namely, GRα and GRβ. Up-regulation of both types of GR isoforms has been observed in the kidney of male mice [27] and nasal polyps [23] in response to GC treatment. While the role of GRα is well characterized, GRβ remains elusive. An association between GC insensitivity and increased GRβ expression has been reported in asthma, rheumatoid arthritis, and inflammatory bowel disease [18], [28], [29]. In addition, some studies have shown that the content and translocation of GRs might be related to GC sensitivity [28]. GRα has a widespread distribution and is responsible for the induction and repression of target genes, while GRβ has been proposed to act as a dominant negative regulator [19]. Therefore, the ratio of GRα/GRβ expression is likely to be important to the GC responsiveness of various cells and tissues, and consistent with this model, GC resistance is associated with low GRα/GRβ ratios [30]. Although both receptors are broadly distributed throughout the cellular framework of the inner ear, a clear relationship between the functional interplay and GRα/GRβ expression has not been established in the human auditory system [31]. We were only able to analyze the total level of GR protein in this study, as specific antibodies against GRα or GRβ of guinea pig are not commercially available. Therefore, we were not able to specifically address which isoform of GR is predominately up-regulated in PBMCs and cochleae after administration of dexamethasone. Nevertheless, our observation of a positive correlation between PBMC GR and the population of cochlear GRs suggests that PBMC GR level might be a useful surrogate indicator for predicting cochlear GR level. In addition to GRs, mineralocorticoid receptors can also play important roles in GC signaling in cochleae [32]. Therefore, future studies that differentiate between the transcriptional expression levels, mutational status, and translocation of GRα and GRβ and mineralocorticoid receptors in the cochlea may further enhance our ability to design and implement effective therapeutic strategies for treating SSNHL.
